# Development of human white matter pathways in utero over the second and third trimester

**DOI:** 10.1073/pnas.2023598118

**Published:** 2021-05-10

**Authors:** Siân Wilson, Maximilian Pietsch, Lucilio Cordero-Grande, Anthony N. Price, Jana Hutter, Jiaxin Xiao, Laura McCabe, Mary A. Rutherford, Emer J. Hughes, Serena J. Counsell, Jacques-Donald Tournier, Tomoki Arichi, Joseph V. Hajnal, A. David Edwards, Daan Christiaens, Jonathan O’Muircheartaigh

**Affiliations:** ^a^Centre for the Developing Brain, School of Biomedical Engineering and Imaging Sciences, King’s College London, London, SE1 7EH, United Kingdom;; ^b^Centre for Neurodevelopmental Disorders, Kings College London, London, SE1 1UL, United Kingdom;; ^c^Biomedical Image Technologies, ETSI Telecomunicación, Universidad Politécnica de Madrid, 28040 Madrid, Spain;; ^d^Biomedical Research Networking Center in Bioengineering, Biomaterials and Nanomedicine (CIBER-BBN), 28029 Madrid, Spain;; ^e^Children’s Neurosciences, Evelina London Children’s Hospital, Guy’s and St Thomas’ NHS Foundation Trust, London SE1 7EH, United Kingdom;; ^f^Department of Bioengineering, Imperial College London, London SW7 2AZ, United Kingdom;; ^g^Department of Electrical Engineering (ESAT/PSI), Katholieke Universiteit Leuven, 3001 Leuven, Belgium;; ^h^Department of Forensic and Neurodevelopmental Sciences, King’s College London, London SE5 8AF, United Kingdom;; ^i^Department of Neuroimaging, Institute of Psychiatry, Psychology and Neuroscience, King’s College London, London SE5 8AF, United Kingdom

**Keywords:** fetal, diffusion MRI, white matter, tractography

## Abstract

During the second and third trimesters of human gestation, rapid neurodevelopment is underpinned by fundamental processes including neuronal migration, cellular organization, cortical layering, and myelination. In this time, white matter growth and maturation lay the foundation for an efficient network of structural connections. Detailed knowledge about this developmental trajectory in the healthy human fetal brain is limited, in part, due to the inherent challenges of acquiring high-quality MRI data from this population. Here, we use state-of-the-art high-resolution multishell motion-corrected diffusion-weighted MRI (dMRI), collected as part of the developing Human Connectome Project (dHCP), to characterize the in utero maturation of white matter microstructure in 113 fetuses aged 22 to 37 wk gestation. We define five major white matter bundles and characterize their microstructural features using both traditional diffusion tensor and multishell multitissue models. We found unique maturational trends in thalamocortical fibers compared with association tracts and identified different maturational trends within specific sections of the corpus callosum. While linear maturational increases in fractional anisotropy were seen in the splenium of the corpus callosum, complex nonlinear trends were seen in the majority of other white matter tracts, with an initial decrease in fractional anisotropy in early gestation followed by a later increase. The latter is of particular interest as it differs markedly from the trends previously described in ex utero preterm infants, suggesting that this normative fetal data can provide significant insights into the abnormalities in connectivity which underlie the neurodevelopmental impairments associated with preterm birth.

## Significance

This work uses state-of-the-art acquisition and analysis methods developed specifically for fetal MRI to delineate the developing brain’s association, projection, and callosal white matter pathways. We describe unique, heterogenous maturational trajectories for different tracts, suggesting that regionally distinct biological mechanisms are at play in building the structural connectome in utero.

In the human fetus, the brain’s major white matter pathways develop over the second to third trimester of gestation in an extremely rapid yet distinctly hierarchical order (1, 2). The structure and the integrity of these white matter connections have an integral role in supporting the efficiency and coordination of functional networks. Current understanding about these processes has been largely reliant on postmortem data (2–6). Fetal MRI can capture whole-brain development in its living, functioning state, thereby providing crucial additional insight into normal growth. In the case of white matter in particular, this can include detailed investigation of developing long-range connections and region-specific trajectories.

The importance of better understanding this key period is emphasized by the high prevalence of cognitive and motor problems in children born preterm. In these infants, early exposure to the ex utero environment likely influences later trajectories of neurodevelopment (7–9). Multiple lines of evidence suggest that white matter abnormalities are the dominant pathology, further suggesting that this specific tissue type is both at a critical stage in its development and vulnerable to external influences (10–15). In this context, characterization of in utero maturation of white matter has a critical role as a normative reference.

Precise characterization of in vivo fetal development of white matter tracts using noninvasive methods such as MRI is challenging due to difficulties inherent to acquiring imaging data from this population, such as addressing image artifacts related to maternal tissue and constant fetal motion, as well as recruiting enough subjects to account for population heterogeneity and age effects (11, 16–27). Previous studies are also difficult to generalize as representing typical development as they have included clinical populations with brain abnormalities or ex utero preterm infants (8, 28, 29). All existing studies have used diffusion-tensor imaging (DTI) to describe changes in microstructure (30); however, the results have been inconsistent. While some studies have reported linear relationships between DTI metrics and gestational age (GA) (16, 22, 23, 31), others have fit nonlinear models (18, 32) and others still have found no clear age-dependence (19, 21).

In this study, we address the limitations of DTI and challenges of fetal imaging using a state-of-the-art high angular resolution multishell diffusion-weighted MRI (dMRI) acquisition, as well as a reconstruction and processing pipeline developed specifically for studying challenging fetal data as part of the developing Human Connectome Project (dHCP) (http://www.developingconnectome.org) (33, 34). We applied newly developed and optimized methods for in utero tractography and microstructure estimation in a large cohort of 113 healthy fetuses from 22 to 37 wk GA. With these methods, we were able to delineate specific white matter bundles including the left and right corticospinal tracts (CST) (an example of a projection tract), the optic radiations (ORs) and inferior longitudinal fasciculus (ILF) (examples of association tracts), and the corpus callosum (CC) (example of a commissural tract). These specific tracts were selected due to known differences in their developmental trajectories and because their injury or abnormal development has been implicated in the pathophysiology of neurodevelopmental disorders or intellectual disability (10, 12, 35). This study represents the largest and most detailed in utero characterization of maturational changes in white matter microstructure across the second to third trimester of human gestation and represents a valuable resource for improving our understanding of the neuropathophysiology underlying neurodevelopmental disorders.

## Results

### Normative Trends for Whole-Brain Growth and FA in the Fetal Cohort.

Fetal dMRI data were collected in 151 subjects (age 22 to 38 wk) as part of the dHCP (details presented in *SI Appendix*, section 1). All fetal brain images were reviewed and reported by an experienced perinatal neuroradiologist as showing appropriate appearances with no evidence of brain injury and/or malformation. Each subject was processed using the dHCP preprocessing pipeline, which includes specific measures to account for the existence of unpredictable fetal motion, geometric distortion of echo planar imaging, signal intensity inhomogeneities caused by differences in fetal position, and poor signal to noise ratio due to the small size of the fetal head and its distance from the coil (36, 37). Of the total 151 subjects that were manually assessed, 38 subjects failed due to excessive motion during acquisition (details of quality checking criteria presented in *SI Appendix*, section 1).

**Fig. 1.**
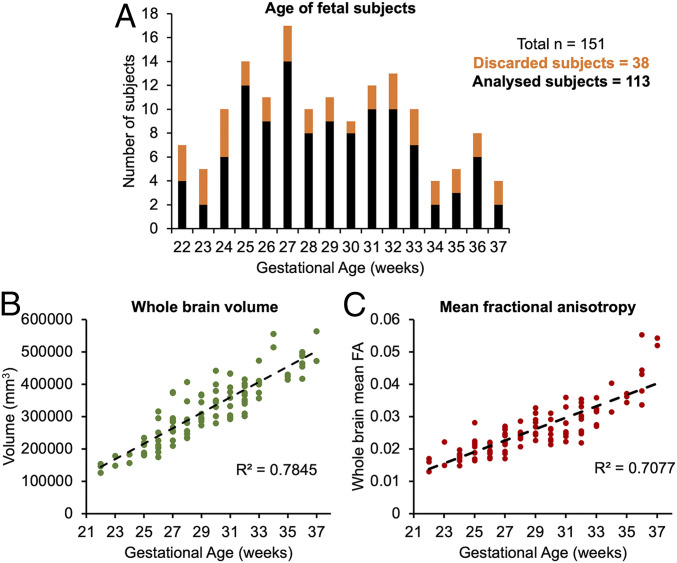
(*A*) Distribution of subjects used in the study according to their GA (black), including those discarded at the tractography stage (orange). (*B*) The whole-brain volume of each subject plotted according to GA. (*C*) The mean FA across the whole brain in each subject, plotted according to GA.

To verify that the data set showed normal expected trends in volumetric growth, we calculated the relationship between whole-brain volume of each subject and GA. Consistent with existing literature, we found a strong linear increase in volume across our study period (R2 = 0.78, *P* < 0.001, Fig. 1*B*) (38). Whole-brain mean FA similarly showed a positive linear relationship with GA (Fig. 1*C*).

### Projection, Association, and Commissural White Matter Microstructure from 22 to 37 wk GA.

**Fig. 2.**
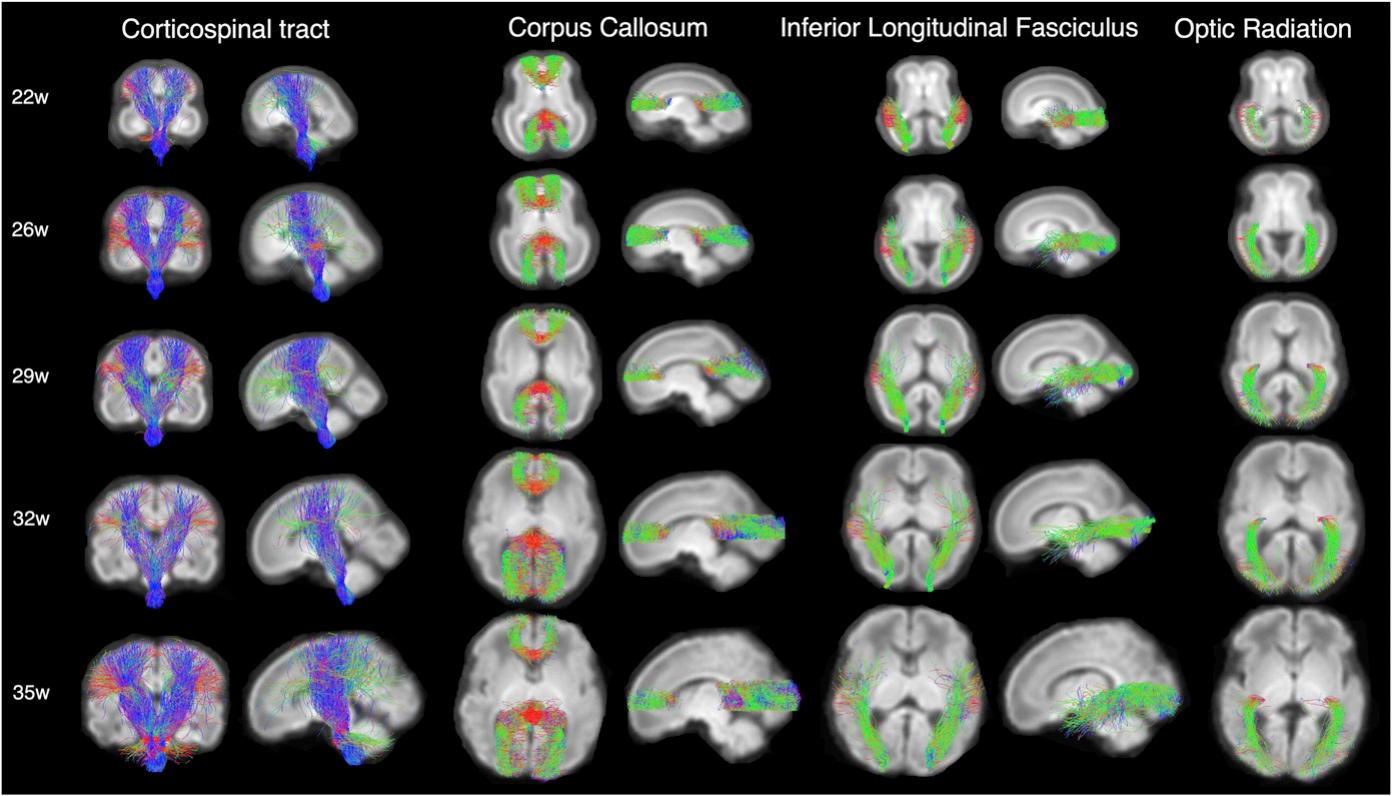
White matter pathways estimated using targeted probabilistic streamline tractography in ODF templates constructed by averaging individual subject ODFs for each gestational week. The color coding of tractography connections is based on a standard red-green-blue code applied to the vector between the end-points of each structure (green for anterior–posterior, red for right–left and blue for dorsal–ventral).

Next, we estimated individual orientation density functions (ODFs) in MRtrix3 using constrained spherical deconvolution (details presented in *SI Appendix*, section 2). This method improves tractography estimations by addressing the challenge of resolving crossing fiber populations within a voxel, which can confound other commonly used methods like diffusion tensor imaging (39, 40). Individual subject ODFs were first compiled into average templates for each gestational week and then probabilistic streamline tractography was used at three-weekly intervals (22, 26, 29, 32, and 35 gestational weeks) to delineate five different white matter pathways (splenium and genu of the CC, CSTs, ILFs, ORs) (regions of interest and paths are described in *SI Appendix*, sections 3.1–3.4). Tractography was successful in all cases with the exception of the OR, which was difficult to estimate in the youngest 22 gestational week template, but could be reliably identified at all other ages (Fig. 2). Template-to-subject warps were then used to transform tracts from the age-matched template to individual subject space (details in *SI Appendix*, section 3).

### White Matter Bundles Have Distinct Maturational Trajectories.

**Fig. 3.**
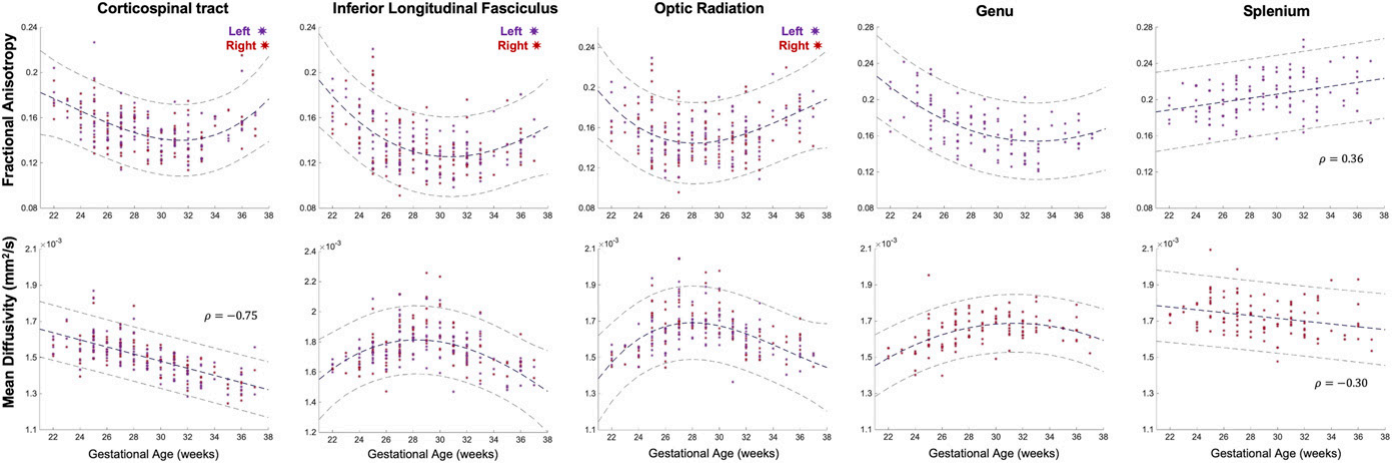
Mean FA and MD values underlying the left (red) and right (purple) CST, ILF, OR, Genu, and splenium for each fetal subject, plotted according to the GA of the subject in weeks. A second degree polynomial curve is fitted for the FA in the CST, ILF, OR, and Genu, the MD in the ILF, OR, and Genu (navy dashed line). The MD in the CST and the FA/MD in the splenium have linear relationships with GA, described by a Spearman’s rank correlation coefficient (ρ). Dashed lines above and below represent the 95% confidence interval.

To place our results in the context of prior studies looking at white matter development, we first used diffusion tensor metrics; FA and mean diffusivity (MD) to estimate changes in the underlying microstructure of each tract. Mean FA and MD showed distinct maturational trajectories within different white matter tracts (Fig. 3). The relationship between GA and tensor metrics was best described by a second degree polynomial fit (as defined by Akaike Information Criterion [AIC]) in the majority of the delineated tracts (Fig. 3), with the exception of FA and MD in the splenium and MD in CST, where the relationship was linear (CST: FA AIC weight (wi) = 0.6; ILF: FA wi = 0.64, MD = 0.70; OR: FA wi = 0.72, MD = 0.73; Genu: FA wi = 0.54, MD wi = 0.71).

As expected, and in keeping with there being complex developmental changes in the white matter across our study period, relationships between FA/MD and GA were significant for all the tracts (*P* < 0.01). There were no significant differences between the left and right hemispheres for any of the delineated tracts (*P* > 0.1). Of particular interest, distinct maturational trends of FA/MD were seen within different sections of the CC, with a linear relationship between FA and GA in the splenium (ρ = 0.36) but a more complex relationship in the genu, with FA values first decreasing from 22 to 30 wk GA and then increasing thereafter toward full term gestation (Fig. 3). The inverse of this relationship was seen in MD values, with a linear decrease in the splenium (ρ = −0.3) and a similar nonlinear relationship in the genu with a peak at ∼30 wk followed by a decline toward term. As with the splenium of the CC, a downward trend in FA from 22 to 30 wk, then a steady incline from 30 wk toward term was identified in the CST, ILF, and OR (Fig. 3). Similarly, inverse trends were seen in these white-matter tracts in the relationship between MD and GA, with an initial rise from 22 to 30 wk followed by a decrease toward full term. The exception was the CST, which showed a strong negative correlation (ρ = −0.75). For completeness, the axial and radial diffusivities underlying each tract were also computed, and these plots can be found in the supplementary information section.

### Validating Tensor Metrics Using Multishell, Multitissue Modeling.

**Fig. 4.**
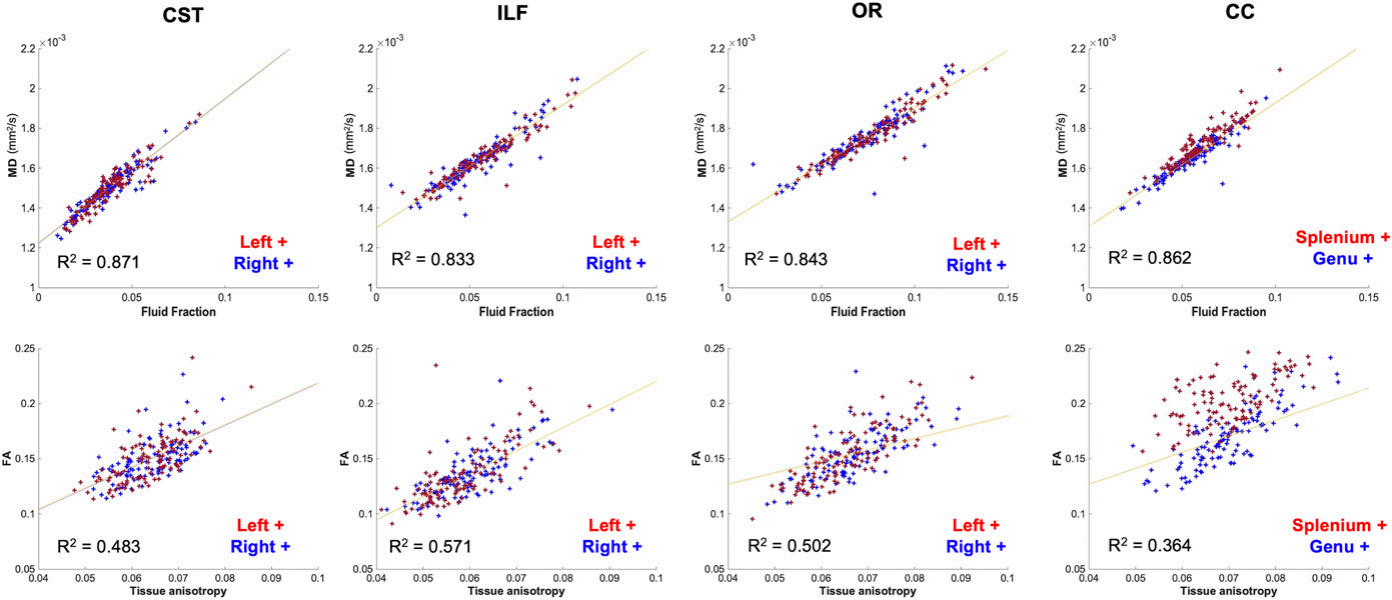
(*Top row*) partial correlations between tract-average MD and volume fraction of the fluid component (significant relationship for all partial correlations, *P* < 0.01). (*Bottom row*) Mean FA value of the tract plotted against the square root of the power in the l = 2 band of the tissue component (tissue anisotropy) values in voxels traversed by each tract (significant relationship for all partial correlations, *P* < 0.05).

Given the relatively small size of the fetal brain, it is plausible that partial-voluming of tissue might have affected the estimated FA and MD values underlying the tracts, especially if streamlines traverse voxels, which contain both white and gray matter or cerebrospinal fluid (CSF) (41). To specifically address these partial-voluming effects and see whether they are responsible for our observed maturational trends, we applied a multishell multitissue constrained spherical deconvolution model to the DWI data (details presented in *SI Appendix*, section 2), which uses the unique b-value dependencies of signal in white matter and CSF to delineate intravoxel contributions of brain tissue and fluid (11, 40). As would be expected, this analysis identified a strong positive linear trend between the fraction of fluid and MD in all of the delineated tracts (Fig. 4) and a positive relationship between mean FA and the tissue anisotropy (Fig. 4). Importantly, these linear trends suggest that the observed nonlinear maturational trends in our data cannot be attributed to simple partial voluming effects. To highlight the similarities between MD and fluid fraction trends over GA, a plot displaying the relationship between fluid fraction and GA can be found in the *SI Appendix*, Fig. S2).

## Discussion

In this work, we used in utero dMRI to report in vivo brain white-matter development in a population of 113 fetuses aged 22 to 37 wk gestation as part of the open-access dHCP. In addition to representing the largest ever cohort of fetuses studied in this way, we used state-of-the-art acquisition and analysis methods, which have enabled the most detailed delineation of the fetal brain’s white-matter pathways to date. Using these methods, we have studied the brain’s major association, projection, and commissural fibers and demonstrate that each have distinct developmental ontogenies, with several showing nonlinear changes in tract microstructure across our study period.

In accordance with postmortem studies describing the presence of commissural and projection fibers as early as the first trimester of gestation, we were able to identify both the genu and the splenium of the CC, the CST, and the ILF in our study population from 22 wk GA onward (Fig. 2) (4, 24, 42–44). Immature axons within the cortico-cortical association tracts have also been seen as early as 15 wk gestation, with the first appearance of the ILF reported around 15 to 17 wk GA (4, 24). In our data, the only white-matter tract which was challenging to delineate at the earliest 22 wk GA time point was the OR, with few streamlines reaching the back of the occipital lobe. This finding is consistent with electron microscopy studies, which report that while the OR is evident from the lateral geniculate nucleus to the subplate by 11 to 13 wk GA (45–47), synapses in the cortical plate are only evident at 23 to 25 wk GA (46, 48).

To provide a comparison with previous studies, which have reported white matter microstructural metrics derived from the widely used tensor model, we quantitively assess changes over the fetal period using FA and MD (30, 49, 50). We observed a linear increase between GA and FA at a whole-brain level, reflecting overall reduction in water content and increasing proportion of white matter relative to gray matter over this timeframe. This simple linear relationship at a whole-brain level highlights that the tracts have unique and distinct trajectories, which are influenced by more complex processes than just overall tissue and fluid balance in the brain. In contrast to our findings, the majority of previous fetal and preterm neonatal diffusion studies have reported linear increases in FA and decreases in MD over their study periods (16, 22, 23, 31). However, many of these study populations have very few subjects under the age of 28 wk, and some have only studied the last trimester of gestation (16, 23, 31). The reported linear increase in FA and decrease in MD in the third trimester in these previous studies therefore corresponds to the changes in FA and MD that we observe in our data between ∼29 and 37 wk in the CST, ILF, OR, and splenium of the CC.

Some prior investigations into fetal white matter development have also found nonlinear relationships between MD and GA (18, 22, 32). As with our results, Schneider et al. found that a second degree polynomial fit best described changes in MD in different white matter regions across the brain (32). Khan et al. also found an increase in MD toward 30 wk gestation followed by a sharp decrease toward term in the cortical plate (22).

The tract-specific maturational trends we observe likely also reflect the known regional differences in white-matter maturation across the brain (2, 51, 52). Tensor metrics are highly sensitive to different biological processes, and therefore the unique trends we observe are likely to have resulted from a combination of multiple factors (29, 53, 54). In our data, a negative linear correlation between MD and GA was seen only in the CST and splenium, which could represent simple reductions in water content and increasing fiber organization in these particular tracts over the third trimester (16, 22, 23, 31). Since histology and ex vivo DTI studies suggest that both the CST and CC tracts are among the earliest to form (24, 42, 55), it is plausible based on this histological evidence that by 22 wk gestation these tracts are already coherently organized and that MD is predominantly being affected by the reduction in water content over our study age range. In contrast, within the ILF, OR, and Genu, the initial increase in MD could be explained by tortuous fibers becoming more coherent over this period, making water diffusion less hindered in the principle direction of the fiber pathway (18). In addition, before 30 wk gestation, there is less tissue organization and there are larger extracellular spaces between fibers to allow for cellular migration, which could further contribute to higher MD values (32). In keeping with this, histological studies have described an abundance of extracellular matrix (ECM) between white-matter fiber bundles in the second trimester, which significantly decreases by 35 wk gestation (56). Since the ECM has high water content, it is possible that its relative abundance around fibers has an effect on MD values and significantly contributes to the trends we observe.

Although FA in older cohorts is often used as a proxy for the degree of integrity or myelination, the white-matter pathways in this study are still relatively immature at birth (57). Biochemical markers of mature myelin (myelin-basic protein) are largely expressed postnatally in the cerebral cortex and then increase substantially in the first 2 y of life (51, 58). However, unmyelinated white-matter tracts still show signal intensity changes consistent with anisotropic water diffusion (59), so it is possible that the increases in FA we observe are due to active axonal outgrowth and initial ensheathment of axons by premyelin sheaths, generated by immature oligodendrocytes (OLs) (58, 60). In agreement with this, OL lineage progression has been shown to affect FA values (61), and the percentage of immature OLs in the cerebral white matter increases markedly from 30 wk gestation (62). Back et al. also identify the first signs of pre-OL ensheathment of axons at ∼30 wk gestation (60), coinciding with the transition in our FA trends for the CST, ILF, OR, and Genu. Of further interest, Xu et al. also identified 30 to 31 wk gestation as a transitional point from high angular resolution diffusion imaging (HARDI)-defined radial coherence to cortico-cortical coherence, indicating the emergence of cortico-cortical association fibers (63). These MRI findings were correlated with histology, where they additionally observed the transformation of radial glial fibers into astrocytes (63). Together, these observations indicate that there are several developmental processes that transition around 30 wk gestation, which are likely to have had an effect on the FA values and are contributing to the unique trends we describe in Fig. 3.

Our results additionally support existing evidence that there is heterogeneity in the development of the different compartments of the CC (51). In our data, the precise neurobiological underpinnings of these differing trajectories between the genu and the splenium cannot be determined just with dMRI data alone. Developing callosal fibers grow through complex pathways and cross the midline using different substrates in transient fetal structures such as the callosal septa. The callosal septa are prominent between 18 and 34 wk, and their biochemical composition is dynamically over this period, including changes in the expression of axonal guidance molecules, cellular matrix, and ECM constituents, which are likely to affect tensor metrics (64, 65). Previous studies have also identified differences in the growth rate of different sections of the CC, with the genu growing at a faster rate than the body and the splenium during fetal development, but then after birth, mature myelination is observed in the splenium before the genu (55, 51, 66, 67). Kinney et al. additionally report a difference in the delay between the onset and maturation of myelin between the anterior and posterior sections of the CC, which might further contribute to the different trends we observe in FA/MD between the two distinct sections. Therefore, it is possible that the initial higher FA values in the genu at 22 wk are reflective of a faster growth rate over the fetal period. The premyelin phase then initiates first in the splenium, resulting in a higher FA in comparison with the genu at full-term age (51). Based on our findings, future studies would benefit from further delineation of the CC into different compartments through combination with histology for more specificity.

Developing white matter is known to be vulnerable to adverse influences related to the extrauterine environment, and early damage leads to significant life-long neurocognitive impairments (7, 68, 69). A comprehensive characterization of in utero normal white matter development is therefore a critically important reference point for comparison with data from preterm infants. In addition to enabling more detailed and reliable tractography, continuing advances in dMRI and processing pipelines can also now provide more information about the underlying microstructural changes. This can give new insight into the mechanisms of white-matter injury, such as why certain tracts appear more susceptible to damage than others and how this is influenced by timing of the related insult (54, 70, 71). In a wider sense, the combination of in utero dMRI with ex utero imaging and histological studies can therefore provide a comprehensive understanding about the role of aberrant early development in the pathophysiology of neurodevelopmental disorders originating in the perinatal period.

To address partial-voluming effects and understand if they could explain the trends seen in our data, we applied a multishell multitissue constrained spherical deconvolution model, which uses the unique b-value dependencies of signal in white matter and CSF to delineate intravoxel contributions of brain tissue and fluid (40, 72). In adults, this more complex approach to modeling voxel-wise diffusion has been shown to improve the precision of fiber orientation estimations at tissue interfaces (73). As seen in neonatal cohorts, partial voluming between tissue and fluid is present in fetal dMRI data to varying degrees throughout the brain and as a function of maturation (34). In the tissue-specific ODFs, we found a strong positive linear relationship in all tracts between the MD value and the fluid fraction, verifying the expected relationship between MD and voxel-wise fluid density. Based on this result, we propose that partial voluming effects alone cannot explain the observed nonlinear U-shaped relationships between GA and FA and MD values.

The image acquisition and processing pipelines used in this study were specifically designed to address the unique challenges associated with fetal and neonatal neuroimaging within the dHCP (http://www.developingconnectome.org/). The advances within this project have both significantly reduced data loss and markedly increased the signal to noise ratio and sensitivity, ultimately offering improved biological interpretation. While there is a large body of literature to support that measuring white-matter structural integrity with DTI has clinical relevance (29, 54, 71, 74), tensor metrics do not provide direct visualization of fiber bundles and therefore findings must be complemented by existing knowledge from histology. However, the noninvasive nature of dMRI allows whole-brain three-dimensional visualization, thus enabling studies investigating the development of long-range connectivity across the entire brain network and comparisons of regional differences in brain development. Through combining state-of-the-art acquisition and methodology, we have sufficient sensitivity to highlight different developmental trajectories within specific white-matter tracts and in doing so provide valuable insights about a fundamental stage in early human brain development.

In summary, we describe the largest study to date in a healthy fetal cohort using dMRI methods to characterize the fundamental processes underlying healthy white-matter development across the late second to third trimesters of human gestation. Our large cohort covers a wide age range and only includes healthy fetuses with no evidence of brain injury, which is in marked contrast to previous reports that have studied narrower windows in development and included fetuses with abnormalities. Our results and the associated data represent a valuable resource, which is being made publicly available, and is representative of healthy white-matter development in utero, which can be compared with that of clinical populations at risk of neurodevelopmental difficulties such as those born preterm.

## Methods

Fetal dMRI data were collected in 151 subjects as part of the dHCP (Fig. 1*A*). Raw data were preprocessed using a bespoke pipeline (details described in *SI Appendix*) that includes denoising, bias correction, dynamic distortion correction, and slice-to-volume motion. Only images that passed quality control were included in this study.

For each subject, the *b* = 0 and *b* = 1,000 volumes were extracted and used to estimate the diffusion tensor and calculate FA/MD maps (details in *SI Appendix*).

We then estimated ODFs for each subject in MRtrix3 (https://www.mrtrix.org/). Individual subject ODFs were compiled into weekly templates (details in *SI Appendix*). Probabilistic streamline tractography was used to estimate the five different tracts; streamlines were guided by specific seed regions, waypoints, and exclusion zones based on the known neuroanatomy of the tracts (details in *SI Appendix*). Tracts were overlaid onto the FA and MD maps, and then the mean FA and MD values were calculated within the overlaid streamlines. The AIC (75) was used to evaluate the most suitable model across different degrees of polynomial fit (1–4) to describe the relationship between GA and FA/MD.

To model the data using a multishell multitissue approach, subject-specific white matter response functions were extracted and the oldest 20 subjects were averaged to obtain a group-average response function of relatively mature white matter (details in *SI Appendix*). A group-average CSF response function was calculated from the whole cohort of subjects. All subjects’ dMRI signals were deconvolved into tissue ODF and fluid components using multishell multitissue constrained spherical deconvolution and the two corresponding group-average response functions. Tracts were overlaid onto the normalized fluid ODF (to approximate the fluid fraction in each voxel) and onto the square root of the power in the l = 2 band of the tissue ODF (representing tissue anisotropy). The mean CSF fraction and mean tissue anisotropy for each tract was calculated.

## Supplementary Material

Supplementary File

## Data Availability

Anonymized open-access fetal MRI data used in this work are available on request and will be released in full as part of the dHCP fetal data release (http://www.developingconnectome.org).

## Change History

May 25, 2021: The license for this article has been updated.
