# Supplementary material for: Development of human white matter pathways in utero over the second and third trimester
Source: Proc Natl Acad Sci U S A. 2021 May 10;118(20):e2023598118. doi: 10.1073/pnas.2023598118 (PMC8157930; doi:10.1073/pnas.2023598118)
Supplement: Supplementary File [file pnas.2023598118.sapp.pdf]

PNAS
www.pnas.org

Supplementary Information for
Regional Development of Human White Matter pathways in utero over the 2nd and 3rd Trimester

Siân Wilson, Maximilian Pietsch, Lucilio Cordero-Grande, Anthony N Price, Jana Hutter, Jiaxin Xiao, Laura McCabe, Mary A Rutherford, Emer J Hughes, Serena J Counsell, Jacques-Donald Tournier, Tomoki Arichi, Joseph V Hajnal, A David Edwards, Daan Christiaens, Jonathan O'Muircheartaigh

Corresponding author: Tomoki Arichi
Email: tomoki.arichi@kcl.ac.uk

This PDF file includes:

Supplementary text
SI References

Supplementary Information

The dHCP data collection and analysis was approved by the UK NHS research ethics committee (REC code: 14/LO/1169). Informed written consent was obtained from each subject prior to participation.

1. Acquisition, pre-processing and quality control. Gestational age was determined by sonography at 12 post-ovulatory weeks as part of their routine clinical care. Fetal MRI data were acquired with a Philips Achieva 3T system with a 32-channel cardiac coil in maternal supine position. dMRI data were collected with a combined spin echo and field echo (SAFE) sequence [1,2] at 2 mm isotropic resolution, using a multi-shell diffusion encoding that consists of 15 volumes at *b*= 0 s/mm^2, 46 volumes at *b*= 400 s/mm^2, and 80 volumes at *b*= 1000 s/mm^2 lasting 14 minutes [3,4]. The protocol also included the collection of structural T2w, T1w, and fMRI data, for a total imaging time of approximately 45 minutes.

Raw data were processed using a bespoke processing and quality assessment pipeline [5] that includes Generalized Singular Value Shrinkage (GSVS) MP-PCA image denoising and Rician bias correction in the acquired magnitude, complex data dynamic distortion correction of susceptibility-induced B0 field changes using the SAFE method [1,6,7] and slice-to-volume motion

correction based on a multi-shell spherical harmonics and radial decomposition (SHARD) representation[5].

Quality control was based on summary metrics designed to detect the amount of motion (based on the gradient of the motion parameters over time) and the percentage of slice dropouts in the data [5]. This was followed up with expert visual assessment, which considered any residual or uncorrected artefacts due to motion, noise and distortion. Of the 151 subjects that were pre-processed, 113 were classified as high quality reconstructions based on the above criteria and were included in this study.

2. Modelling diffusion weighted imaging data. For each subject, the motion and distortion-corrected *b=0* and *b=1000* volumes were extracted and used to estimate the diffusion tensor, using the iteratively reweighted linear least squares estimator in *MRtrix3* [8] and FA/MD maps were calculated [9].

For spatial alignment, orientation density functions (ODFs) were estimated in *MRtrix3* using constrained spherical deconvolution [10,11]. Individual subject ODFs were compiled into weekly templates through a series of coarse pose normalisation and nonlinear diffeomorphic image registration steps [12–14]. The resulting ODF templates were then iteratively nonlinearly aligned to a joint population template using median aggregation [13,14]. The resulting transformations were composed to obtain pairs of inverse consistent diffeomorphic subject-to-template and template-to-subject warps.

For analysis, WM and CSF response functions were estimated for each subject in subject-native space using co-registered age-matched T2-based tissue segmentations [15] as inclusion areas. WM response functions were extracted from the CST and CC using the *tournier* algorithm and CSF responses using the *dhollander* algorithm in *MRtrix3* [8,16]. The WM response functions of the oldest 20 subjects were averaged to obtain a group-average response function of relatively mature WM, a group-average CSF response function was calculated from the whole cohort of subjects. All subjects' dMRI signal was subsequently deconvolved into tissue ODF and scalar fluid components using multi-shell multi-tissue constrained spherical deconvolution [11] and the two corresponding group-average response functions. For each subject, the resulting components were intensity normalised [13] and, to be able to compare FA values with tissue anisotropy, the power spectrum in the tissue component was calculated from the square root of the power in the l=2 band of the spherical harmonics on the normalised ODF. Finally, all normalised quantities were transformed to their respective template-space using the subject-to-template warps.

3. Tractography. The ODF templates were used as a guideline for selecting regions of interest (ROIs) to perform tractography. Targeted probabilistic streamline tractography as implemented in *MRtrix3*, was performed in 5 different weekly templates (at intervals of ~3 weeks) on the normalised age-averaged tissue ODFs to delineate the CST, ILF, OR and CC (Figure 2). This approach was used in order to maintain a standardised region of interest for calculating microstructural metrics. Tractography was performed independently in the left and right hemispheres of each template, due to the recorded hemispheric asymmetry of the fetal brain [17]. Tracts were defined based on multiple spatially independent ROIs, originating in seed regions, with at least one further specific inclusion zone and with exclusion zones to limit tracts to a single hemisphere if appropriate (specific details of how each tract was delineated are described below). Template-to-subject warps were constructed using the FNIRT package in FSL [18] and used to transform tracts from the age-matched template to individual subject space. Tracts were sifted for spurious streamlines using tcksift [8,19].

3.1 Cortico-spinal tract (CST). For tracking both the left and right CST, the cerebral peduncle of the lower brain stem was used as a seed region, with waypoints in the left and right posterior limb of the internal capsule (PLIC). Both masks were drawn using axial brain slices and were 3 slices thick. The left and right primary motor cortices, were used as respective tract endpoint regions.

The central sulcus was used as a reference point to identify this region in the 3D-volumetric reconstruction of the brain. An angulation threshold of 15° was used.

3.2 Inferior lateral fasciculus (ILF). Seed-region masks were placed in coronal slices in the left and right anterior temporal lobes, with a waypoint mask placed along part of the lateral and inferior wall of the lateral ventricle. To avoid inclusion of fibres crossing the midline, an exclusion zone was placed through the corpus callosum with a sagittal mask along the midline. Angular threshold of 20° was used.

3.3 Optic radiation (OR). The left and right optic radiations were tracked separately, using the left and right lateral geniculate nucleus as a seed region. A waypoint was drawn in coronal slices, in the posterior occipital lobe, estimating the location of the primary visual cortex. The exclusion zone consisted of a multi-slice sagittal mask along the midline and a multi-slice axial mask immediately superior to the corpus callosum. Angular threshold of 20° was used.

3.4 Corpus callosum (CC). The genu and the splenium of the corpus callosum were tracked independently, due to an *ex utero* autopsy study reporting different sequences of myelination in the subregions of the corpus callosum [20]. Exclusion zones were placed in axial slices above and below the corpus callosum and in mid-coronal slices. Due to the curvature of the tracts, angulation thresholds were set higher at 45°.

4. Analysis of microstructural change metrics. Diffusion tensor model: Tracts were overlaid onto the FA and MD maps and then the mean FA and MD values were calculated within the overlaid streamlines. Multi-shell multi-tissue constrained spherical deconvolution: Tracts were overlaid onto the normalised fluid ODF, and onto the square root of the power in the l=2 band of the tissue ODF, representing tissue anisotropy and the mean value within the streamlines was calculated.

5. Statistical modelling. Akaike information criterion (AIC) was used to evaluate the most suitable model across different degrees of polynomial fit (1-4), to describe the relationship between gestational age and FA/MD [21]. This was determined according to the lowest AIC value and highest rounded Akaike weight (w_i). Where a linear trend was identified, Spearman's rank correlation coefficients (ρ) were calculated to illustrate the strength and direction of the association between the mean FA/MD of each tract and gestational age of the subject.

SI References

1. Hutter, J. *et al.* Quiet echo planar imaging for functional and diffusion MRI. *Magn. Reson. Med.* **79**, 1447–1459 (2018).
2. Ferrazzi, G. *et al.* An efficient sequence for fetal brain imaging at 3T with enhanced T1 contrast and motion robustness. *Magn. Reson. Med.* **80**, 137–146 (2018).
3. Tournier, J. *et al.* A data-driven approach to optimising the encoding for multi-shell diffusion MRI with application to neonatal imaging. *NMR Biomed.* **33**, (2020).
4. Hutter, J. *et al.* Slice-level diffusion encoding for motion and distortion correction. *Med. Image Anal.* **48**, 214–229 (2018).
5. Christiaens, D. *et al.* Scattered slice SHARD reconstruction for motion correction in multi-shell diffusion MRI. *Neuroimage* **225**, 117437 (2021).
6. Cordero-Grande, L., Christiaens, D., Hutter, J., Price, A. N. & Hajnal, J. V. Complex diffusion-weighted image estimation via matrix recovery under general noise models. *Neuroimage* **200**, 391–404 (2019).
7. Ghiglia, D. C. & Romero, L. A. Robust two-dimensional weighted and unweighted phase unwrapping that uses fast transforms and iterative methods. *J. Opt. Soc. Am. A* **11**, 107 (1994).
8. Tournier, J. D. *et al.* MRtrix3: A fast, flexible and open software framework for medical image processing and visualisation. *NeuroImage* vol. 202 (2019).
9. Basser, P. J., Mattiello, J. & Lebihan, D. Estimation of the Effective Self-Diffusion Tensor from the NMR Spin Echo. *J. Magn. Reson. Ser. B* **103**, 247–254 (1994).

10. Tournier, J. D., Calamante, F. & Connelly, A. Robust determination of the fibre orientation distribution in diffusion MRI: Non-negativity constrained super-resolved spherical deconvolution. *Neuroimage* **35**, 1459–1472 (2007).
11. Jeurissen, B., Tournier, J. D., Dhollander, T., Connelly, A. & Sijbers, J. Multi-tissue constrained spherical deconvolution for improved analysis of multi-shell diffusion MRI data. *Neuroimage* **103**, 411–426 (2014).
12. Jenkinson, M., Bannister, P., Brady, M. & Smith, S. Improved optimization for the robust and accurate linear registration and motion correction of brain images. *Neuroimage* **17**, 825–841 (2002).
13. Raffelt, D. *et al.* Symmetric diffeomorphic registration of fibre orientation distributions. *Neuroimage* **56**, 1171–1180 (2011).
14. Pietsch, M. *et al.* A framework for multi-component analysis of diffusion MRI data over the neonatal period. *Neuroimage* **186**, 321–337 (2019).
15. Serag, A. *et al. A. SERAG ET AL.: A 4D PROBABILISTIC ATLAS OF THE DEVELOPING BRAIN A Multi-channel 4D Probabilistic Atlas of the Developing Brain: Application to Fetuses and Neonates*. *Annals of the BMVA* vol. 2012 www.brain-development.org. (2012).
16. Tournier, J. D., Calamante, F. & Connelly, A. Determination of the appropriate b value and number of gradient directions for high-angular-resolution diffusion-weighted imaging. *NMR Biomed.* **26**, 1775–1786 (2013).
17. Bracco, L., Tiezzi, A., Ginanneschi, A., Campanella, C. & Amaducci, L. Lateralization of choline acetyltransferase (ChAT) activity in fetus and adult human brain. *Neurosci. Lett.* **50**, 301–305 (1984).
18. Jenkinson, M., Beckmann, C. F., Behrens, T. E. J., Woolrich, M. W. & Smith, S. M. Review FSL. *Neuroimage* **62**, 782–790 (2012).
19. Tournier, J.-D., Calamante, F. & Connelly, A. MRtrix: Diffusion tractography in crossing fiber regions. *Int. J. Imaging Syst. Technol.* **22**, 53–66 (2012).
20. Kinney, H. C., Brody, B. A., Kloman, A. S. & Gilles, F. H. Sequence of central nervous system myelination in human infancy: II. Patterns of myelination in autopsied infants. *J. Neuropathol. Exp. Neurol.* **47**, 217–234 (1988).
21. Akaike, H. Information theory and an extension of the maximum likelihood principle. Proceedings of the 2nd international symposium on information theory. *Second Int. Symp. Inf. Theory* (1973).

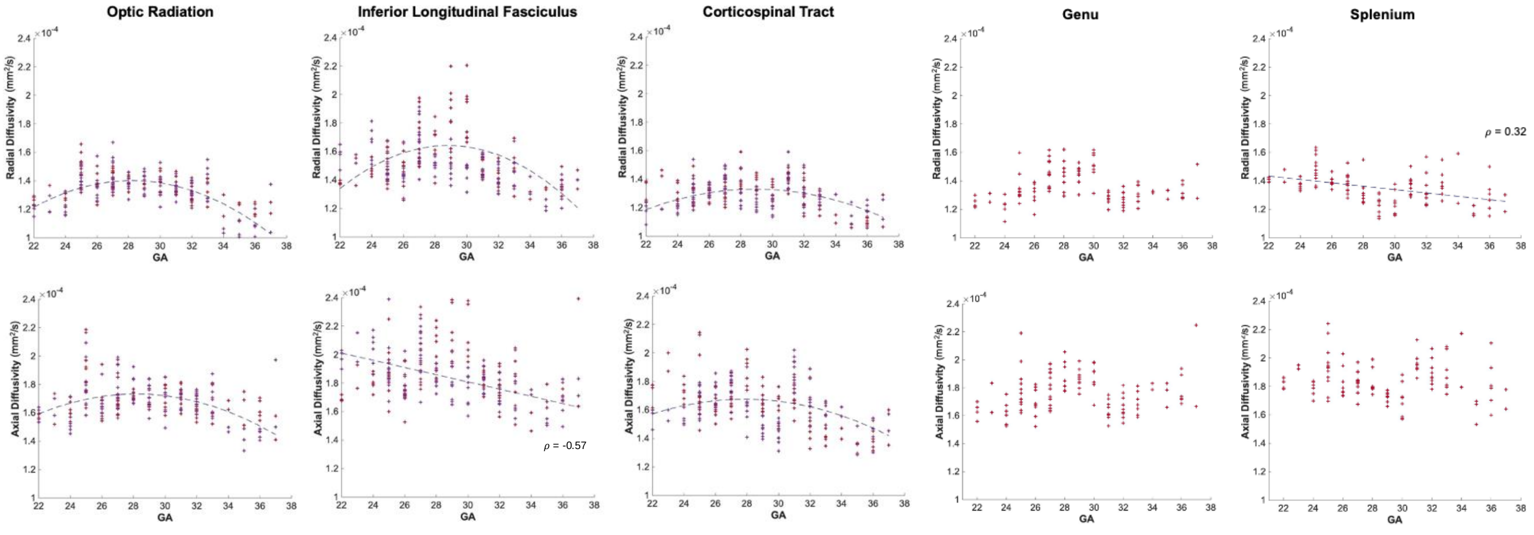

Figure S1. Axial (AD) and radial diffusivities (RD) for each tract over gestational age. Mean AD and RD values underlying the left (red) and right (purple) CST, ILF, OR, Genu and Splenium for

each fetal subject, plotted according to the gestational age (GA) of the subject in weeks. A second degree polynomial curve is fitted for the AD in the CST and OR, the RD in the CST, ILF, and OR (navy dashed line) ($p < 0.05$). The AD in the ILF and the RD in the splenium have linear relationships with gestational age, described by a Spearman's rank correlation coefficient (ρ), ($p < 0.05$). No significant relationship found between AD and GA in the splenium and genu.

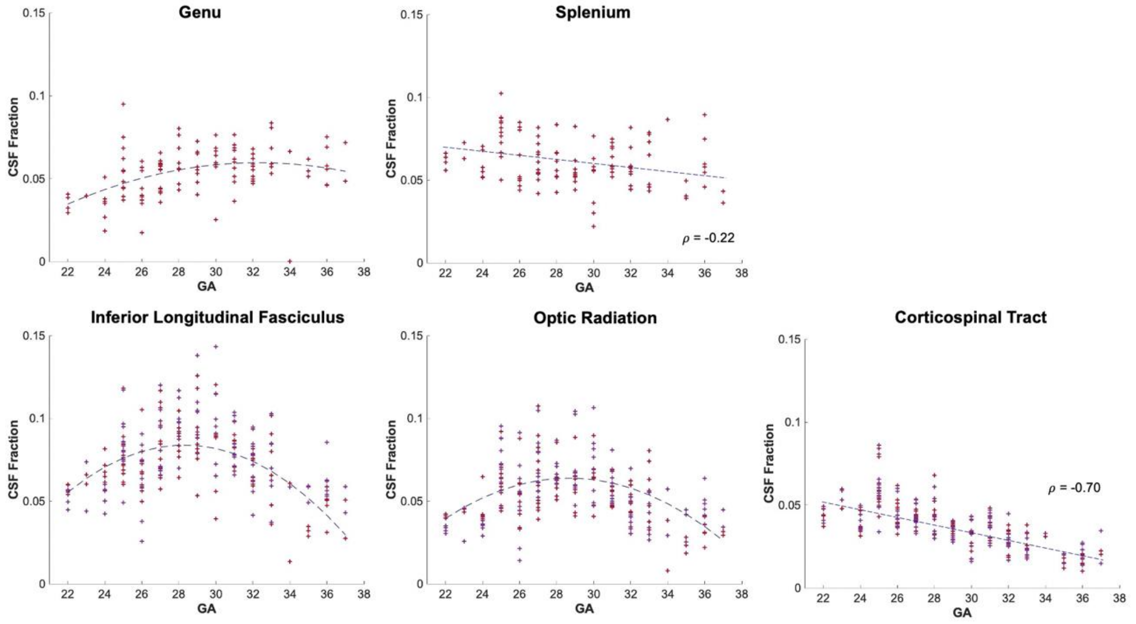

Figure S2. Fluid fraction changes with gestational age. Linear relationships between CSF fraction and GA in the splenium and CST, quantified using Spearman's rank correlations (ρ). In the Genu, ILF, and OR, 2nd degree polynomial trends were fit (according to AIC).
